# Respiratory Syncytial Virus Persistence in Macrophages Upregulates Fcgamma Receptors Expression

**DOI:** 10.3390/v6020624

**Published:** 2014-02-06

**Authors:** Jorge Gaona, Carlos Santiago-Olivares, Enrique Ortega, Beatriz Gómez

**Affiliations:** 1 Department of Microbiology and Parasitology, Faculty of Medicine, National Autonomous University of Mexico (UNAM), Ciudad Universitaria, D.F. Mexico C.P. 04510, Mexico; E-Mails: jgaber2007@gmail.com (J.G.); carlosantiagolivares@yahoo.com.mx (C.S.-O.); 2 Department of Immunology, Biomedical Research Institute, National Autonomous University of Mexico (UNAM), Ciudad Universitaria, D.F. México C.P. 04510, Mexico; E-Mail: ortsoto@unam.mx

**Keywords:** RSV persistence, macrophages, FcγRs expression

## Abstract

Viruses can persist in differentiated cells (*i.e.*, macrophages) over long periods of time, altering host cells functions but not inducing their death. We had previously reported that, in early passages (14–40) of a murine macrophage-like cell line persistently infected with respiratory syncytial virus (RSV) (MɸP), FcγR-mediated phagocytosis and expression of FcγRIIB/RIII on the cell membrane were increased with respect to mock-infected macrophages (MɸN). In this work, we explored the mechanism underlying such effects. Increases in FcγR expression and FcγR-mediated phagocytosis are preserved after more than 87 passages of the persistently infected culture. We analyzed the expression of FcγR isoforms at both mRNA and protein levels, and found out that RSV persistence distinctly affects the expression of FcγR isoforms. We also observed that the increase in FcγRs expression results neither from soluble factors (cytokines) or viral products released by the infected cells, nor from an increase in the rate of FcγR internalization. Our results suggest that RSV persistence in macrophages induce intracellular effects that have an impact on FcγRs gene expression at both mRNA and protein levels, and that the characteristics of RSV persistence were preserved for over 87 passages.

## 1. Introduction

Viruses persisting in differentiated cells—*i.e.*, macrophages (Mɸs)—can regulate the expression of both their own genes and those of the host cell in order to achieve residence in a non-lytic state, besides selectively affecting functions of the infected cell without destroying it [1]. Mɸs play very important roles in innate and adaptive immune responses since they are involved in various processes, such as phagocytosis, antigen presentation, and cytokine production [2,3]. Mɸs express on their membrane receptors for the Fc region of IgG antibodies (FcγRs). Alterations of FcγRs-mediated phagocytosis *in vitro* and *in vivo* have been reported in Mɸs persistently infected with an RNA virus (HIV-1). This effect is caused by a decreased synthesis of the gamma chain of receptors, suggesting that viral persistence alters gene expression in the host cell [4,5]. Changes in host cell-gene expression resulting from persistence of respiratory syncytial virus (RSV), another RNA virus, have also been reported: in the human epithelial cell line HEp-2, viral persistence alters the expression of host genes involved in cell survival and chemokine production [6]. Also, we had previously shown that, in a macrophage-like murine cell line persistently infected with RSV (MɸP) [7], FcγRs expression, FcγRs-mediated phagocytosis, and cytokines production are altered [8].

Cell-surface FcγRs comprise a family of integral membrane glycoproteins expressed by most leukocytes. Murine Mɸs express four different FcγRs: while FcγRI, FcγRIII, and FcγRIV are activatory, FcγRIIB mediates the inhibition of FcγR- mediated signals [9,10]. Aggregation of FcγRs by antigen-antibody complexes or IgG-opsonized particles triggers several effects or functions, including phagocytosis [11]. FcγRs have also been shown to participate in both the afferent phase of immune responses and immune system homeostasis [12,13].

RSV, a paramyxovirus of the genus *Pneumovirus*, is prevalent and highly infectious [14]. Worldwide, it is a very important pathogen that causes frequent acute upper and lower respiratory tract infections, especially among infants and young children [15,16]. RSV is also a paramount cause of morbidity and mortality in the elderly and in immunocompromised patients [17], constituting the second leading cause of death due to viral infections in elderly individuals [18]. While seasonal RSV outbreaks occur each year globally, the RSV virus can be detected only during the winter epidemic season [14,19]. Young children who have recovered from severe RSV bronchiolitis often develop chronic and recurrent respiratory problems [20,21]. The link between RSV infection, the development of sequelae (wheezing, asthma) [22,23] and chronic obstructive pulmonary disease has been clearly established in several well-controlled prospective epidemiological studies [24,25], suggesting that RSV persists in individuals with this condition [26]. The delayed effects of severe RSV disease might be partially explained by viral persistence, which may cause chronic inflammation [26,27] and/or change cell genome expression patterns.

Persistent infection with RSV occurs in patients with T-cell immunodeficiencies, and establishing persistent infections in tissue culture of either epithelial or hematopoietic cells (e.g., HEp-2 [6] or Mɸ [7]) is easy. RSV persistence in animal models has been reported in infected nude mice [28,29,30], which develop chronic airway function abnormalities [31,32,33]. Persistence of bovine respiratory syncytial virus, which is closely related to RSV, has also been demonstrated in B cells from naturally infected cows [34]. Although viral persistence in humans has not been clearly proven, circumstantial evidence suggests its occurrence. Immunohistological observations indicate the presence of RSV antigens in osteoclasts and in multinucleated cells formed in bone marrow cultures from patients with Paget’s disease [35]. In addition, RSV nucleic acid was detected in archival postmortem lung tissue from infants who had died during the summertime, without apparent clinical disease having been reported, suggesting that the virus might persist in lungs after an acute infection [36]. Since no animal reservoir of RSV has been found, persistent human infections by RSV may be implicated, at least partially, in preserving the virus during inter-epidemic periods. Despite the evidence that RSV is able to establish persistent infections in various cell types, and the possible relationship between RSV persistence and human disease [23,24,25,26,37], few studies have investigated the effects that persistent infection by this virus can have on the functions of infected cells.

We have previously reported that persistent infection by RSV in a murine macrophage-like cell line (MɸP) alters the expression of FcγRIIB/RIII on the cell membrane and FcγRs-mediated phagocytosis [8]. In this work, we explored the mechanism underlying such effects, studying the effect of RSV persistence on the expression of the different FcγR isoforms at both mRNA and protein level, and determining whether the effect is caused by soluble factors (cytokines) or viral products released by infected cells.

## 2. Results and Discussion

### 2.1. Results

#### 2.1.1. Persistent Infection by RSV Increases FcγR-mediated Phagocytosis and FcγRIIB/RIII Expression even at High Passage Numbers

**Figure 1:**
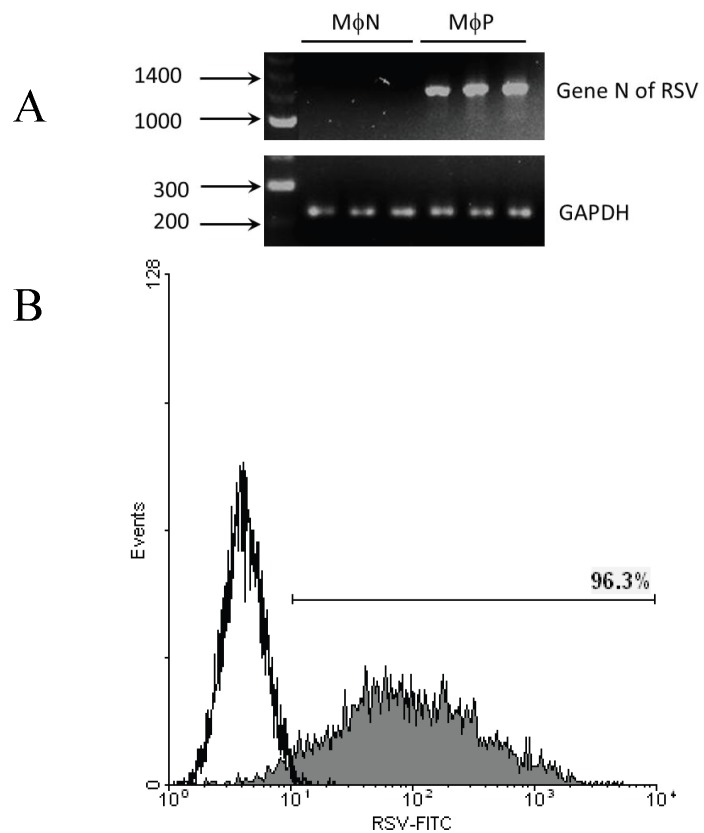
RSV persistence in MɸP. (**A**) Presence of mRNA of the N gene in MɸP. Agarose/EtBr gel (2%) electrophoresis of RT-PCR products. Total RNA from three different passages of MɸP (72, 83 and 87) or MɸN cells was harvested and converted to cDNA. DNA primers for the N gene were used to amplify a segment of 1,187 bp by PCR. As control, a segment of RNA for GAPDH was amplified; (**B**) Expression of RSV antigens on MɸP membrane. MɸN (empty histogram) or MɸP (gray histogram) were stained with FITC-labeled anti RSV antibodies and analyzed by flow cytometry. Histogram is representative of several experiments with different passages of MɸP and MɸN during the course of these experiments.

Throughout this paper, we refer to MɸP as cells of the murine macrophage-like cell line (P388D1) persistently infected with RSV, and to MɸN as mock-infected P388D1 cells. Persistent infection in the MɸP cultures used in these experiments was confirmed by the presence of mRNA for the N gene and the expression of viral antigens in 92%–96% of cells (Figure 1). Viral persistence parameters (infective units in culture supernatants and % of RSV antigen-positive cells) did not differ significantly from earlier reported values [7].

**Figure 2:**
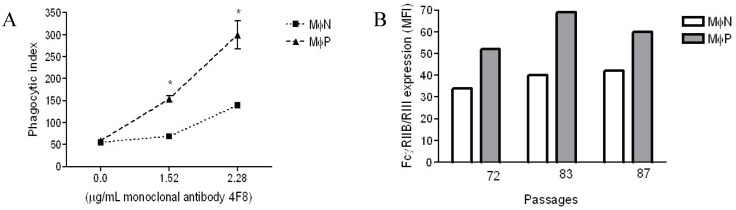
FcγRIIB/RIII mediated phagocytic activity and expression of FcγRIIB/RIII receptors in MɸP and MɸN. (**A**) Phagocytosis of IgG-opsonized and non-opsonized SRBC by MɸN and MɸP. Cells were incubated with 2,4,6-trinitrophenyl-labeled SRBC opsonized with the indicated concentrations of the anti-2,4-dinitrophenol (anti-DNP) IgG antibody 4F8. Results are expressed as mean ± 1 SD from three independent experiments passages of MɸN and passages 72, 83 and 87 of MɸP (* *p <* 0.05); (**B**) Expression of FcγRIIB/RIII in the above mentioned passages of MɸN or MɸP cells. Cell-membrane FcγRIIB/RIII were stained with specific monoclonal antibody 2.4G2 and secondary FITC-labeled F(ab')_2_ fragments of anti-rat antibodies, and analyzed by flow cytometry. Each individual bar represents the MFI of 10,000 cells. Statistical analysis of the average of the three different passages of MɸN and MɸP (38.67 ± 2.4 *vs*. 60.33 ± 4.9, respectively) indicates a significant difference in FcγRIIB/RIII expression (*p* = 0.016).

Previously, we had reported that in MɸP early passages (14–40), FcγRs-mediated phagocytosis and plasma membrane expression of FcγRIIB/RIII increase in comparison with non-infected MɸN [8]. In this work, we evaluated whether the same characteristics were present in MɸP later culture passages (72 to 87). Phagocytosis and expression of FcγRIIB/RIII were determined as previously described [8]. We found out that, after more than 70–80 passages, MɸP still showed higher levels of phagocytic activity and cell surface FcγRIIB/RIII expression than MɸN cells (Figure 2). As expected, phagocytic activity was dependent on the IgG concentration used for opsonization, and the phagocytosis of IgG-opsonized sheep red blood cells (SRBCs) by MɸP was always significantly higher (usually by a factor of 2) than that of MɸN. It is noteworthy that no differences were observed in the phagocytosis of non-opsonized erythrocytes, which suggests that the effect is characteristic of FcγR-mediated phagocytosis (Figure 2A). Increases in the expression of cell surface FcγRIIB/RIII in MɸP (50%–72%) (Figure 2B) was similar to those previously reported for early culture passages [8].

#### 2.1.2. Increase in FcγRIIB/RIII Cell Membrane Expression Is Not Mediated by Soluble Factors

**Figure 3:**
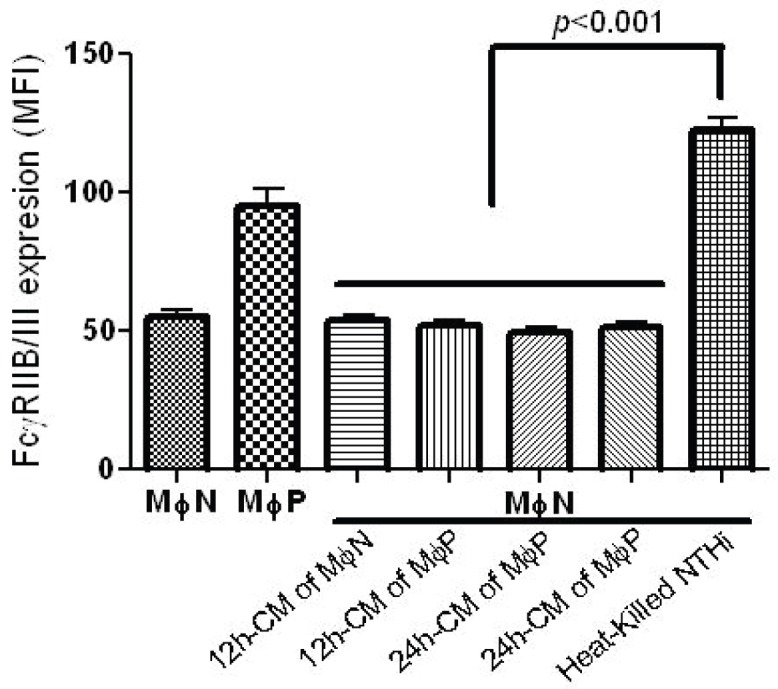
FcγRIIB/RIII expression in MɸN after treatment with MɸN or MɸP conditioned medium (CM). MɸN were treated with either 12 h- or 24 h-CM from MɸN or MɸP, or with heat-killed NTHi as control (see Materials and Methods for details). After 24 h, FcγRIIB/RIII was analyzed by flow cytometry. Results are expressed as mean ± 1 SD of mean fluorescence intensity in three independent experiments.

Persistence of RSV in macrophages could induce the release of extracellular factors (e.g., cytokines, viral particles, viral products), which would act in a paracrine-like way in order to induce the increase in FcγRIIB/RIII expression. Seeking to investigate whether the increased expression of FcγRIIB/RIII was produced by factors released by persistently infected MɸP, we treated MɸN cells with a conditioned medium obtained from MɸN or MɸP cultures after an incubation period of 12 h or 24 h. Expression levels of FcγRIIB/RIII were determined by flow cytometry. Mean fluorescence intensity (MFI) of FcγRIIB/RIII expression by MɸN was not significantly altered when MɸN were incubated for 24 h with supernatants from MɸN or MɸP (Figure 3). So as to verify that MɸN are able to increase the expression of FcγRIIB/III in response to a stimulus already known to increase the expression of these receptors [38], MɸN were incubated with heat-killed NHTi, which induced a significant increase in FcγR expression as compared to cells treated with conditioned medium (Figure 3). This suggests that the MɸN cell line is able to respond to an activating stimulus. These results show that the increase in the expression of FcγRIIB/III induced by RSV persistence is not mediated by extracellular factors released by persistently infected cells. 

#### 2.1.3. RSV Persistence does not Affect FcγRIIB/RIII Endocytosis

**Figure 4:**
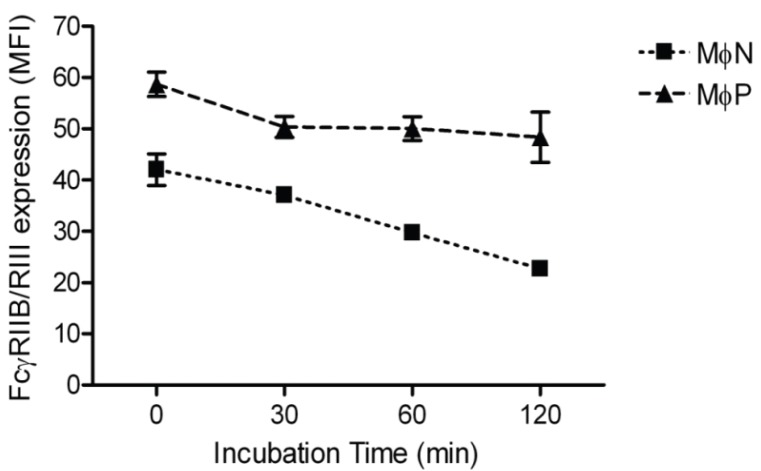
Internalization of FcγRIIB/RIII in MɸN and MɸP. Internalization kinetics of mAb 2.4G2-FcγRIIB/RIII complexes was monitored by flow cytometry during 120 min, as described in Materials and Methods. Results are expressed as the mean ± 1 SD from three independent experiments. No significant difference was observed between MɸN and MɸP in the net amount of internalized mAb 2.4G2-FcγRIIB/RIII complexes.

In order to determine whether the increase in membrane FcγRIIB/RIII was due to impaired receptor endocytosis, we measured the rate of FcγRIIB/RIII internalization as described in Materials and Methods. We found similar FcγRs internalization kinetics in both MɸN and MɸP. Average decreases in MFI at 120 min were 14.82 and 15.79 units for MɸN and MɸP, respectively, suggesting that FcγRIIB/RIII receptors endocytosis is not significantly altered by RSV persistence (Figure 4).

#### 2.1.4. Intracellular Levels of FcγRIIB/RIII Proteins Are Increased in MɸP

**Figure 5:**
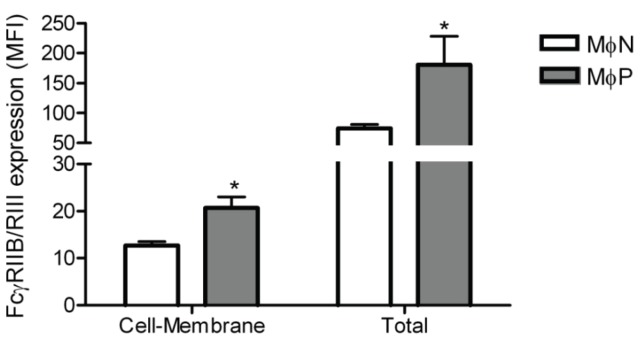
Total FcγRIIB/RIII protein in MɸN and MɸP. Cell-membrane and total FcγRIIB/RIII protein content were determined in non-permeabilized or permeabilized MɸN and MɸP cells. FcγRIIB/RIII expression was evaluated with 2.4G2 monoclonal antibody and analyzed by flow cytometry. Results are expressed as mean ± 1 SD from three independent experiments, using different passages of MɸN and MɸP.* indicates *p* < 0.05.

In order to investigate whether the increase of FcγRIIB/RIII expression on the membrane of MɸPs was associated with increased receptor synthesis, we determined the total amount of receptor protein (membrane and intracellular) in the cells using flow cytometry. We found out that MɸPs have more FcγRIIB/RIII protein than MɸNs, both on the cell surface and intracellularly (Figure 5). These results suggest that viral persistence induces the upregulation of FcγRIIB/RIII synthesis.

#### 2.1.5. Membrane Expression of FcγRIIB and FcγRIII, but Not of FcγRI, Is Increased in RSV Persistently Infected Cells

Four different types of murine Fcγ receptors have been described (RI to RIV). In our initial work we measured the expression of FcγRs with mAb 2.4G2, which recognizes both FcγRIIB and FcγRIII [39]. In order to determine the effect of RSV persistence on the expression levels of FcγRI, FcγRIIB and FcγRIII separately, we compared the amount of each protein in MɸPs and MɸNs lysates by Western blot using antibodies specific for each isoform. Lack of a suitable commercial antibody prevented us from determining FcγRIV.

**Figure 6:**
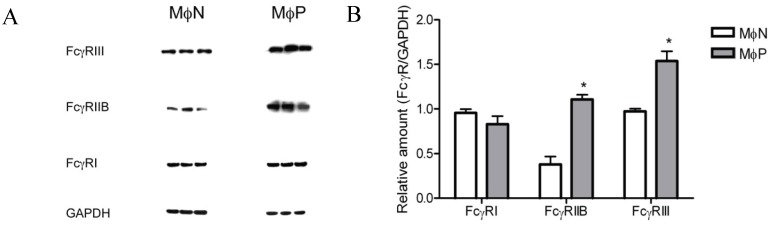
Total FcγRI, RIIB, and RIII protein in MɸN and MɸP cell extracts. (**A**) Cells from three different passages of MɸN and cells from passages 72, 83 and 87 of MɸP were lysed and FcγRI, FcγRIIB, FcγRIII and GAPDH proteins in cell extracts were determined by Western blot with specific antibodies; (**B**) The relative amount of each FcγR isoform was determined as the ratio of the densitometric intensity of the FcγR band to the intensity of the GAPDH band in the correspondent cell extract. Results are expressed as mean ± 1 SD (* indicates *p* < 0.05).

The protein content of FcγRIIB and FcγRIII was observed to be higher in MɸP than in MɸN: fold increases were 1.9 and 0.6, respectively. In contrast, no significant difference was found in FcγRI levels between in MɸP and MɸN (Figure 6). It is interesting that the highest effect of RSV persistence was the increase in FcγRIIB, an inhibitory receptor, and yet phagocytosis is higher in MɸPs cells. This discrepancy might be related to the IgG isotype used for opsonization (IgG2b), which is very weakly recognized by FcγRIIB [40].

#### 2.1.6. mRNA Expression of FcγRs Is Distinctively Affected by Viral Persistence

**Figure 7:**
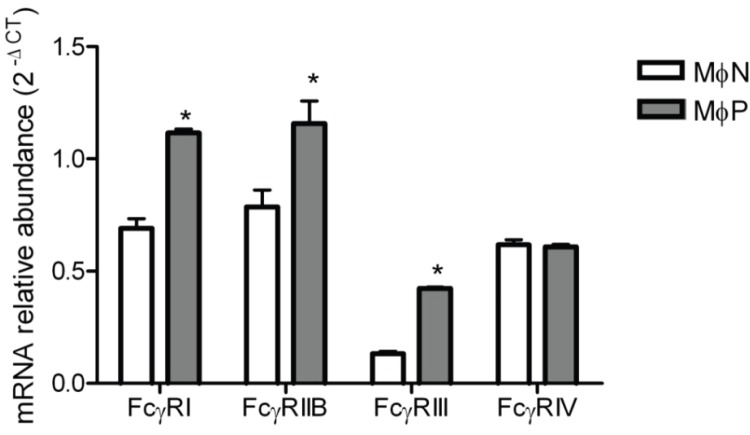
FcγRs mRNA expression in MɸN and MɸP. Total RNA from MɸN and MɸP cells was harvested and converted to cDNA, and mRNAs for FcγRI, RIIB, RIII and RIV were measured by real-time PCR. Transcript levels were normalized to GAPDH mRNA expression. Data are expressed as 2^−ΔCT^. Results are shown as mean ± 1 SD from three independent experiments using cells from passages 72, 83 and 87, * indicates *p* < 0.05.

Since we could not evaluate the effect of RSV persistence on the expression of FcγRIV by Western blot, and we wanted to have a better understanding of the effect of RSV persistence on the expression of FcγRs, we resorted to RT-PCR so as to determine mRNA levels for all four FcγR types in MɸPs and MɸNs. Results are shown in Figure 7. Levels of mRNA for FcγRIV were similar in MɸNs and MɸPs, suggesting that RSV persistence has no effect in the expression levels of this receptor. As expected from the results of Western blots, mRNAs for FcγRIIB and FcγRIII were higher in MɸPs than in MɸNs, with increases of 0.4 and 2.2 fold, respectively. Unexpectedly, we found that the FcγRI mRNA was also increased (0.6 fold) in MɸPs with respect to MɸNs, which suggests that this receptor’s expression level is also regulated at the post-transcriptional level. 

### 2.2. Discussion

Viral persistence in cell lines is a well established model that can be used to study alterations in the expression profile of the host genome, which are caused by the constant expression of viral genes [1]. We and other researchers have shown that RSV persistence in cell cultures alters the profile of host genome expression. RSV persistence has been reported in various cell lines, and in two of them, HEp-2, (human epithelial cell line) [6] and P338D1 (murine macrophage-like cell line) [7], the characteristics resulting from persistent viral infection have been extensively studied. Although these two cell models of RSV persistence share several characteristics, they differ in some others. Thus, for instance, expression of viral antigens in RSV-infected HEp-2 cells ranges from nil to highly positive, a high titer of extracellular virus is produced, and syncytia are formed [6]. In contrast, we have found that the characteristics of viral persistence in an RSV-infected murine macrophage-like cell line, MɸP (derived from P338D1), are different: 92%–96% of the cells express viral antigens—as determined by flow cytometry—low titers of extracellular infective virus are produced, and no syncytia are observed. These characteristics are still present after 87 passages. Also, RSV persistence produces an increase in the expression of FcγRs and phagocytic activity in MɸP as compared to MɸN. RSV persistence induced no cell activation, as evaluated by nitrite production. However, it is clear that RSV persistence induces alterations in the expression of host genes, as demonstrated by the downregulation of ICAM-1 [41] and upregulation of membrane receptors (FcγRs [8]), cytokines [8,42] and chemokines [43]. In addition, expression of the anti-apoptotic proteins Bcl-2, Bcl-X, and XIAP was enhanced, while Bcl-X and XIAP were regulated post-transcriptionally [44]. 

In this work, we focused on exploring the mechanisms possibly responsible for the increase in FcγR-mediated phagocytosis that we had previously reported to occur in early passages (14–40) of the persistently infected macrophage-like cell line MɸP [8]. This increase is still evident after 87 passages (Figure 2). The likelihood that the observed effect on FcγRs expression resulted from an autocrine/paracrine-like action of factors (*i.e*., cytokines, viral particles, viral proteins) released by infected cells was ruled out, because extracellular components in conditioned medium from MɸP did not alter FcγRIIB/RIII expression in MɸN (Figure 3). This suggests that the higher expression of FcγRIIB/RIII seen in MɸP is not due to stimulation either by cytokines (IL-1β, IL-6, [8]) or chemokines (RANTES, MIP-1α, MIP-1β, MIP-2; Tirado R. personal communication), which have been detected in MɸP supernatants. We also analyzed the possibility that the observed increase in membrane expression of FcγRIIB/RIII resulted from a reduced rate of FcγR internalization induced by RSV persistence. However, we found no difference between MɸN and MɸP in the kinetics of FcγRIIB /RIII internalization (Figure 4).

Since neither endocytic activity nor components of the conditioned medium (viral particles, viral proteins, cytokines, chemokines, *etc*.) seemed to participate in the increased FcγRs expression, we hypothesized that the observed effect of viral persistence was caused by RSV inducing an increase in the synthesis of FcγRs. Results obtained by flow cytometry assays of cell-membrane and intracellular protein expression (Figure 5) were consistent with our hypothesis. Since mouse cells express four different FcγR isoforms, and because up to this point we had only evaluated the expression of FcγRIIB and FcγRIII isoforms with a monoclonal antibody which recognizes both, we set out to determine the effect of RSV persistence in the expression of each of the four FcγR isoforms expressed by mouse macrophages (FcγRI, FcγRIIB, FcγRIII and FcγRIV) by Western blot with isoform-specific antibodies and by quantitative RT-PCR. We found out that RSV persistence distinctively affects expression of FcγR isoforms. Protein levels of FcγRIIB and FcγRIII are increased in MɸP as compared to MɸN, while FcγRI levels are not affected. FcγRIV levels could not be determined because no suitable commercial antibody is available at present. However, since the levels of mRNA for FcγRIV were not altered, it is reasonable to expect that the membrane expression of this isoform is not altered either. Higher protein levels of FcγRIIB and FcγRIII are consistent with the increased levels of mRNA for these two isoforms.

The effect of the observed changes in the expression of FcγR isoforms on the activation of effector functions of macrophages, is expected to depend on the IgG isotype involved [40]. IgG1 isotype has a higher affinity for the inhibitory FcγRIIB isoform than for the activatory FcγRIII and FcγRIV isoforms. Thus, the increases in FcγRIIB and FcγRIII expression would be expected to result in a lower cell response to immune complexes or opsonized particles containing IgG1. In contrast, IgG2a and IgG2b show higher affinity for the activatory FcγRIII and FcγRIV isoforms than for FcγRIIB. Therefore, effector functions mediated by these isotypes are less sensitive to changes in FcγRIIB. In fact, this is consistent with our observation that FcγR-mediated phagocytosis of IgG2b-opsonized erythrocytes is increased in MɸP. However, it should be kept in mind that phagocytosis is a complex cellular process, involving many proteins and signaling molecules—thus, it is not expected to depend solely on the expression of the receptor involved. For example, FcγRs-mediated phagocytosis in human monocyte-derived macrophages (MDM) increases after IL-10 treatment and decreases after IFN-γ treatment, but these opposite effects do not correlate with the changes in FcγR expression induced by these cytokines [45]. The effect of RSV persistence on the expression of FcγRs mRNA suggests that RSV persistence is able to affect transcription factors, such as PU.1, which participates in FcγRs transcription [46]. Further work is underway in our laboratory in order to examine the participation of PU.1 in FcγRs transcription in RSV infected cells.

It is interesting that, while RSV-induced changes in protein levels of FcγRIIB and FcγRIII correlated with changes in the respective mRNA levels, FcγRI protein expression levels were not significantly affected by RSV persistence, although its mRNA was found to be increased. This suggests that expression of cellular genes in MɸP can be regulated at either the transcriptional and/or post-transcriptional levels. Consistent with this notion are studies reporting that the expression of transmembrane protein ICAM-1 is transcriptionally downregulated [41]. 

Changes in FcγRs expression in macrophages persistently infected with RSV, besides its effect on phagocytosis, could affect other effector functions mediated by these receptors, such as ADCC. However, as previously mentioned, effects of the observed changes are also expected to be highly influenced by the antibody isotypes involved. RSV persistence could also have an impact on the release of inflammatory mediators, thus affecting the functions of macrophages and other cells involved in host defense, although RSV persistence does not seem to alter endogenous or exogenous viral antigen presentation [42]. Additional studies are needed to further characterize the effects of RSV persistence in macrophages. Finally, it is important to study the effects of RSV persistence in human cells in order to evaluate the possible significance of these results to human disease, since differences have been reported in immune cells between mice and human [47].

## 3. Experimental Section

### 3.1. Virus and Cell Lines

The RSV Long strain wild type (wt RSV) has been the prototype virus used in our laboratory. Both the virus origin and the procedures for propagating, purifying and measuring its infectivity in Vero cells have been previously reported [7]. The murine macrophage-like cell line P388D1 was originally obtained from ATCC (TIB 63; Rockville, MD, USA). A sub-line of P388D1 cells persistently infected with wt RSV (MɸP) was obtained in our laboratory by infecting the original cell line with RSV at a multiplicity of infection (moi) of 1, and then culturing the surviving cells [7]. As control, P388D1 cells were mock-infected and subcultured in parallel conditions (MɸN). Both cell lines are maintained by subculturing. MɸP passages from 72 to 87 were used in this study. Throughout the passages, the presence of viral genome was monitored by detecting the mRNA of the gene N by conventional RT-PCR, using a primer pair to amplify a segment of 1,187 bp between nucleotides 1,140–2,327: forward 5'-ATGGCTCTTAGCAAAGTC-3', Reverse 5'-TTTTTTGTTAACTTCAAGCTCTACATC-3'. A segment of 260 bp of GAPDH was amplified as control. RSV antigen was detected by flow cytometry on the surface of 92%–97% of the MɸP using FITC-labeled polyclonal anti-RSV (Oxoid, Hampshire, UK), diluted 1:10 in PBS containing 0.1% (w/v) BSA (PBSA) (Figure 1). Extracellular viral infectivity titer was 1–2 × 10^2^ TCID_50_/mL per 1–2 × 10^6^ cells, and no syncytia were observed [7]. 

In order to verify lack of activation, the production of nitrates by MɸN and MɸP was routinely evaluated by the Griess reaction. Nitrate concentrations in supernatants usually ranged from: 7.1–8.0 μmol/mL per 5–6 × 10^6^ cells for MɸN and 8.3–10.2 μmol/mL per 5–6 × 10^6^ cells for MɸP.

Both cultures were propagated in RPMI 1640 medium (GIBCO/BRL, Grand Island, NY, USA) supplemented with 0.2% NaHCO_3_, 10 mM HEPES, 1 μM 2-mercaptoethanol, and 10% heat-inactivated (56 °C; 30 min) fetal bovine serum (FBS) (Biowest, Veracruz, Mexico). 

### 3.2. Phagocytosis Assays

Phagocytosis of non-opsonized and IgG-opsonized sheep red blood cells (IgG-SRBC) was performed as previously described [8,48], but with minor modifications. In brief, SRBC were labeled with trinitrobenzene-sulfonic acid (TNBS, Sigma Aldrich Corp., St. Louis, MO, USA) 1 mg/mL for 30 min at room temperature. After washing, SRBCs were opsonized by incubation with one out of two different sub-hemagglutinating concentrations (1.52 or 2.28 μg/mL) of affinity-purified murine monoclonal anti-DNP IgG (4F8; IgG2b, produced in our laboratory). Concentrations higher than 2.28 μg/mL could not be used due to agglutination of erythrocytes.

For phagocytosis assays, MɸN or MɸP were plated in 96-well plates (1 × 10^5^ cells/200 μL of RPMI per well). Non-opsonized or opsonized SRBC (25 μL/well of a 2% suspension of SRBC) were added, and the plates were incubated for 60 min at 37 °C. Non-ingested SRBC were lysed with distilled water, and the cells were exhaustively washed with PBS. Cells in the wells were lysed with sodium dodecyl sulfate (1% in PBS), and the pseudoperoxidase activity of the ingested SRBC hemoglobin was determined by a colorimetric assay using 3,3'-diaminobenzidine as substrate. Optical density was read at 492 nm. Results are expressed as phagocytic index = (number of cells *×* Optical Density/100). In all experiments, phagocytosis of opsonized and non-opsonized SRBC was determined in sextuplicate wells. Controls for endogenous enzymatic activity consisted in lysates of six additional wells to which no SRBC had been added, treated under the same conditions.

### 3.3. Flow Cytometry

Cell surface expression of FcγRs was determined by flow cytometry using anti-FcγRIIB/III (2.4G2, a rat monoclonal antibody that recognizes FcγRIIB and RIII epitopes) as unconjugated primary antibody and goat anti-rat F(ab')_2_-FITC as secondary antibody, both from Santa Cruz Biotechnology (Santa Cruz, CA, USA). Cells (5 × 10^5^) from different MɸP or MɸN passages were washed with PBSA and fixed with 4% paraformaldehyde (30 min; room temperature). Fixed cells were washed with PBSA and incubated in PBS + 1% BSA containing 15 μg of 2.4G2 antibody (2 h; room temperature). After incubation, cells were washed twice with PBSA, and then incubated with goat anti-rat F(ab')_2_-FITC (1 h; at room temperature and protected from light). Cells were resuspended in PBS (300 μL), and 10,000 cells were analyzed by flow cytometry (FACScan, Becton Dickinson, Mountain View, CA, USA). So as to determine intracellular proteins, cells were fixed and permeabilized with cold methanol:acetone, 1:1 (1 min; room temperature). Afterwards, cells were washed with PBSA and incubated in PBS + 1% BSA containing 10 μg of 2.4G2 antibody (2 h; room temperature). Following that incubation, cells were washed twice with PBSA and then incubated with goat anti-rat F(ab')_2_-FITC (1 h; at room temperature and protected from light). Thereafter, cells were resuspended in 300 μL of PBS and analyzed by flow cytometry.

### 3.4. Conditioned Medium

Confluent MɸN or MɸP cultures (approx. 5 × 10^6^ cells) were washed with PBS, and then seven mL of supplemented RPMI were added. After 12 or 24 h of incubation at 37 °C, supernatants were collected and cellular debris was removed by centrifugation (352 ×*g*). (We know that cytokine concentrations in supernatants reach a plateau at 24 h [43]). Two mL of this conditioned medium were added to MɸN cultures (1 × 10^6^ cells) and the cultures were incubated for 24 h at 37 °C, 5% CO_2_ atmosphere. As positive stimulation control, MɸN were incubated with heat-killed non-typeable *Haemophilus influenzae* (NTHi) strain 2019 (moi of 100), kindly provided by Dr. Michael Apicella (Department of Microbiology, College of Medicine, University of Iowa, Iowa City, IA, USA). NTHi had been heat-killed (60 min; 70 °C water bath), washed twice by centrifugation (20 min; 900 ×*g*; 4 °C), and suspended in PBS at a final concentration of 1 × 10^10^ bacteria/mL. The expression of FcγRIIB/RIII was determined by flow cytometry, as described above.

### 3.5. Endocytosis Assay

The endocytosis of cell-membrane FcγRIIB/RIII receptors was monitored with the monoclonal antibody 2.4G2. Briefly, 2.4G2 antibody was added to MɸN or MɸP (5 × 10^5^) cells at 20 μg/mL (30 min, 4 °C). After this period, cells were washed thoroughly with ice-cold medium without FBS. Endocytosis was started by adding medium at 37 °C and then incubating at 37 °C for different periods of time (0, 30, 60, and 120 min). Thereafter, cells were fixed with 4% paraformaldehyde (30 min; room temperature) and washed. Afterwards, F(ab')_2_-FITC against the primary antibody 2.4G2 was added. Fluorescence intensity was determined by flow cytometry as described above.

### 3.6. Western Blot

Whole-cell extracts were prepared from MɸN or MɸP (3 × 10^6^ cells) with ice-cold lysing buffer (50 mM Tris-HCl, pH 7.4, 150 mM NaCl, 1% Triton X-100, 0.5% sodium deoxycholate, and 0.1% SDS) containing 1× protease inhibitors cocktail (Sigma Aldrich Corp., St. Louis, MO, USA). After incubation (10 min on ice), lysates were collected and detergent-insoluble material was removed by centrifugation (20 min, 4 °C; 8,800 ×*g*). Protein concentration was determined using the DC protein assay kit (Bio-Rad, Hercules, CA, USA). Samples containing 20 μg of protein each were then boiled in 4 × SDS sample buffer and resolved on NovexBis-Tris Mini Gels NuPAGE (Invitrogen, Carlsbad, CA, USA). Subsequently, proteins were transferred to PVDF membranes (Amersham, Piscataway, NJ, USA) and nonspecific binding sites were blocked by immersing the membranes in blocking solution (PBS, 0.1% Tween-20, and 5% low-fat milk) (1 h, room temperature). After washing the membranes with PBS-0.1% Tween-20, they were incubated with the primary antibody diluted 1:500 in blocking solution (4 °C, overnight), followed by a peroxidase-conjugated appropriate secondary antibody (Santa Cruz Biotechnology, CA, USA), diluted 1:2,500 in blocking solution (1 h, room temperature). The primary antibodies used for Western blot were anti-FcγRI goat polyclonal antibody, anti-FcγRIIB rabbit polyclonal antibody, anti-FcγRIII mouse monoclonal antibody (clone ASH1975), and anti-GAPDH, all from Santa Cruz Biotechnology. Proteins were detected using a chemiluminescent substrate, Super Signal West Dura Extended Duration substrate (Pierce Thermo Scientific, Rockford, IL, USA), and their intensities were normalized to that of GAPDH. Densitometric analysis of results was performed in images obtained with the Chemidox XRS (BioRad, Hercules, CA, USA) and analyzed with the Quantity one software (Bio-Rad).

### 3.7. Real Time RT-PCR

Total RNA was extracted from MɸN or MɸP (2 × 10^6^ cells) with TRIzol, following the manufacturer’s instructions. RNA (2 μg) was reverse transcribed with RNA transcriptase Superscript II (Invitrogen, Carlsbad, CA, USA). TaqMan real-time PCR was performed with primers and probes (assay on demand 20× mix) for FcγRs genes and GAPDH as control gene, using TaqMan assay reagent master mix (Applied Biosystems, Foster City, CA, USA). Real-time PCR was performed with 80 ng of cDNA for both target genes and endogenous control. Cycling parameters were established according to the manufacturer’s protocol. Triplicate CT values were analyzed in Microsoft Excel, using the comparative CT (ΔT) method as described by the manufacturer (Applied Biosystems, Branchburg, NJ, USA). The amount of mRNA from each target gene (2^−ΔCT^) was obtained by normalizing it to the endogenous reference (GAPDH) sample.

### 3.8. Statistics

Data are expressed as mean ± 1 SD from the indicated number of experiments. Differences between groups were determined by Student’s *t*-test, using GraphPad Prism 4.0 software (GraphPad, San Diego CA, USA), and considered statistically significant at *p* < 0.05.

## 4. Conclusions

RSV persistent infection in a murine macrophage-like cell line alters FcγR expression and induces an increase in FcγR-mediated phagocytosis. Increases in FcγR expression and FcγR-mediated phagocytosis are preserved after more than 87 passages of the persistently infected culture. The increase in FcγRs expression results neither from soluble factors (cytokines) or viral products released by the infected cells, nor from an increase in the rate of FcγR internalization. Persistence of RSV genome in infected cells distinctly affects the expression of FcγR isoforms at both the mRNA and protein levels. These results indicate that RSV persistence, a phenomenon possibly more common than usually assumed, might affect the expression of cellular genes and, consequently, normal cell functions as well.
